# Morphometric and Genetic Differentiation of Two Sibling Gossamer-Wing Damselflies, *Euphaea formosa* and *E. yayeyamana*, and Adaptive Trait Divergence in Subtropical East Asian Islands

**DOI:** 10.1673/031.012.5301

**Published:** 2012-04-17

**Authors:** Yat-Hung Lee, Chung-Ping Lin

**Affiliations:** Department of Life Sciences and Center for Tropical Ecology and Biodiversity, Tunghai University, Taichung, 40704, Taiwan

**Keywords:** body size, insular adaptation, lriomote, Ishigaki, Taiwan, wing shape

## Abstract

Insular species frequently demonstrate different tendencies to become smaller or larger than their continental relatives. Two sibling gossamer—wing damselflies, *Euphaea formosa* (Odonata: Euphaeidae) from Taiwan and *E. yayeyamana* from the Yaeyama Islands of Japan, have no clear structural differentiation, and can only be recognized by their geographical distribution, sizes, and subtle differences in wing shape and coloration. This study combined morphometric and genetic techniques to investigate the adaptive significance of trait divergence and species status in these two *Euphaea* damselflies. Phylogenetic analyses of the mitochondrial *cox2* sequences demonstrated that the two damselflies are monophyletic lineages and constitute valid phylogenetic species. The landmark—based geometric morphometrics indicated that the two damselflies are different morphological species characterized by distinctive wing shapes. The larger *E. formosa* exhibited broader hind wings, whereas *E. yayeyamana* had narrower and elongated forewings. The body size and wing shape variations among populations of the two species do not follow the expected pattern of neutral evolution, suggesting that the evolutionary divergence of these two traits is likely to be subjected to natural or sexual selection. The decreased body size, elongated forewings, and narrower hind wings of *E. yayeyamana* may represent insular adaptation to limited resources and reduced territorial competition on smaller islands.

## Introduction

Oceanic islands are considered excellent natural laboratories, and for many decades they have provided scientists with a range of simplified and replicated “natural experiments” for studying ecological and evolutionary processes (Wallace 1880; Darwin 1909; MacArthur and Wilson 1967; Carlquist, 1974; Grant 1986). Body size change and loss of dispersal ability are two well—known ecogeographical patterns among island species (Lomolino et al. 2005; Whittaker and Fernández-Palacios 2007). Insular species frequently demonstrate different tendencies to become smaller (dwarfism in larger species) or larger (gigantism in smaller species) than their close continental relatives (‘the island rule’; Foster 1964; Van Valen 1973; Lomolino et al. 2005). Once they have successfully colonized isolated islands, insular species may reduce flying capacity or develop into flightless forms owing to limited food resources or ecological release (decreased predation and competition) (McNab 1994).

**Figure 1.**
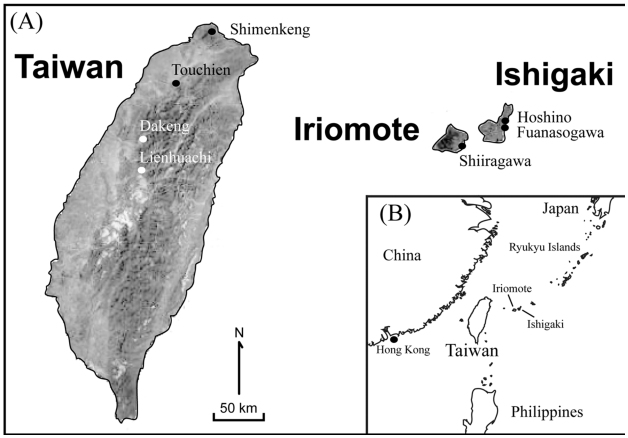
Sampling localities and external morphology of *Euphaea formosa* and *Euphaea yayeyamana*. (A) Present map of Taiwan, lriomote, and lshigaki highlighting sampling sites for damselflies used in this study. (B) Map of subtropical East Asian islands. High quality figures are available online.

**Figure 2.**
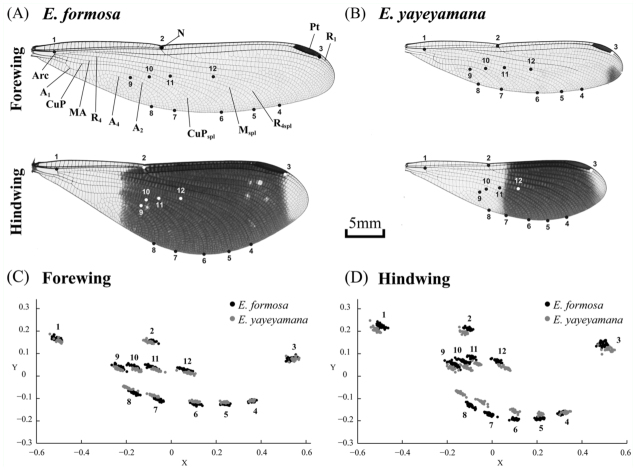
Locality of 12 landmarks used to define wing shapes in (A) *Euphaea formosa* and (B) *Euphaea yayeyamana*: (1) anterior end of the Arculus (Arc); (2) the Nodus (N); (3) posterior intersection of the Pterostigma and Radius 1 (R_1_); (4) posterior end of the Radius 4 (R_4_); (5) posterior end of the Anterior Media (MA); (6) posterior end of the Cubital Vein (CuP); (7) posterior end of the Anal Vein 1 (A_1_); (8) posterior end of the Anal Vein 4 (A_4_); (9) anterior end of the Anal Vein 2 (A_2_); (10) anterior end of the Cubital Vein Supplementary (Cup_spl_); (11) anterior end of the Anterior Media Supplementary (M_spl_); and (12) anterior end of the Radius 4 Supplementary (R4_spl_). CuP, Cubital vein; Pt, Pterostigma. The wing vein nomenclature was modified from Tillyard and Fraser (1940). Scatter plots of Procrustes shape coordinates of (C) forewings and (D) hind wings of 30 *E. formosa* and 27 *E. yayeyamana* individuals. High quality figures are available online.

*Euphaea formosa* Hagen (Odonata: Euphaeidae) and *E. yayeyamana* Matsumura and Oguma are two morphologically similar gossamer—wing damselflies endemic to Taiwan and the Yaeyama (Iriomote and Ishigaki) Islands of Japan, respectively (Matsuki and Lien 1978, 1984; Hayashi 1990; Ozono et al. 2007) (Figures 1A and 1B). Body size reduction in *E. yayeyamana* (dwarfism) compared with *E. formosa* was hypothesized to result from lower prey availability in streams of the smaller Iriomote and Ishigaki Islands than on mainland Taiwan (Hayashi 1990). In addition to body size differences, the overall shape of forewings and hind wings of male *E. yayeyamana* appears to be narrower than that of *E. formosa* (Figures 2A and 2B). The shape of insect wings can largely determine the energetic costs and maneuverability of flight (Betts and Wootton 1988; Grodnitsky 1999; Dudley 2000; Wooton and Kukalová-Peck 2000). Therefore, wing shape differences in these two *Euphaea* damselflies are expected to be optimized by selection for flight performance, which is likely related to their foraging strategies, dispersal abilities (Hayashi 1990), food and predation stress (Stoks 2001; Svensson and Friberg 2007), or sexual environment (Outomuro and Johansson 2011). In addition to selection, changes in body size and wing shape of insular species can arise from random evolutionary processes including genetic drift, the founder effect, and population bottlenecks (Lomolino et al. 2005; Whittaker and Fernández-Palacios 2007). The relative effectiveness of stochastic and selective processes for generating phenotypic differentiation in natural populations is still a matter of debate (Clegg et al. 2002; Hankison and Ptacek 2008). The roles of genetic drift, gene flow, and selection in shaping species differentiation can be assessed by comparing phenotypic variation among populations to that in neutral genetic markers (Clegg et al. 2002; Ahrens and Ribera 2009). Concordant population divergence in neutral genetic markers and phenotypic traits would suggest that random evolutionary mechanisms are responsible for generating the population— specific variations. Conversely, discordant divergence in neutral genetic markers and phenotypic traits would imply that selective forces determine trait variations among populations.

The gossamer—wing damselfly genus *Euphaea* comprises 30 recognized species distributed in tropical and subtropical Asia (Schorr and Paulson 2009). They are medium-sized damselflies occurring predominately in lower to middle elevational forest streams (Orr and Hämäläinen 2003). All *Euphaea* species are territorial, and males aggressively defend their perching sites of emerged vegetation or rocks and exhibit aggressive behavior towards intruding conspecific males. Females appear periodically inside these territories and mate with territory owners. Males of several *Euphaea* species have extensive metallic colors and patches of dark pigments on the hind wings, whereas females are cryptic brownish with transparent wings (Orr and Hämäläinen 2003). The males of *E. formosa* and *E. yayeyamana* are characterized by metallic brown or black patches on the hind wings and distinct red stripes on the thorax. Unlike other congeneric species inhabiting the Asian continent, these island—dwelling *Euphaea* species are more abundant on open streams without thick canopy cover (Hayashi 1990; Huang and Lin 2011). Currently these two closely related endemic *Euphaea* damselflies are designated as separate species on the basis of geographical distribution (Matsuki and Lien 1978; Ozono et al. 2007). However, the most commonly used character system for species designation, the male genitalia, provides no useful structural characteristics for distinguishing between the two species (Matsuki and Lien 1984; Hayashi 1990). An earlier study comparing external morphological characters of *E. formosa* and *E. yayeyamana* demonstrated no distinct differentiation except that *E. yayeyamana* is smaller (Hayashi 1990). Nevertheless, males of the two *Euphaea* species differ in terms of wing pigmentation. *Euphaea yayeyamana* has a small, pigmented patch near the distal edge of the forewing, whereas the forewing of *E. formosa* is hyaline (Matsuki and Lien 1984; Ozono et al. 2007) (Figures 2A and 2B). In addition, *E. formosa* has a more widespread pigmented patch on the hind wing than *E. yayeyamana*. Adult body size and coloration of aquatic insects at maturity vary considerably depending on larval nutrients and environmental parameters of the microhabitats including temperature and water level (Nylin and Gotthard 1998; Corbet 1999). Therefore, the designation of species status for these two *Euphaea* species based solely on sizes and coloration is not adequate, and additional characteristics from other independent sources, such as multiple landmarks in a morphometric analysis or genetic data, are required.

The present study was designed to test three specific hypotheses: (1) Two *Euphaea* damselflies differ in the shape of the forewings and hind wings; (2) the two *Euphaea* damselflies are distinct morphological, genetic, and phylogenetic species; (3) selection operates on body size and wing shape variations of the two *Euphaea* damselflies. In this study, landmark—based geometric morphometric methods (Rohlf and Marcus 1993; Zelditch et al. 2004) and phylogenetic analyses of mitochondrial DNA sequences were combined to determine whether *E. formosa* and *E. yayeyamana* differ in wing shape and form genetically distinguishable lineages. The level of body size, wing shape, and genetic differentiation among geographic populations of these *Euphaea* damselflies were compared to detect the presence of directional or stabilizing selection on the wings. Any sign of selection on wing shape probably reflects evolutionary changes in flight performance and dispersal ability during island evolution.

## Materials and Methods

### Collection, DNA extraction, and sequencing

A total of 30 *E. formosa* and 27 *E. yayeyamana* males were collected from around Taiwan and the Ishigaki and Iromote Islands of Japan, respectively (Figure 1A, Table 1). Damselfly specimens of two out— group species, *E. decorata* Hagen in Selys and *E. ornata* (Campion) were collected from Tai Po Kau of Hong Kong and Mt. Diaoluo of Hainan Island, respectively, for phylogenetic analyses (Figure 1B, Table 1). All insect specimens were preserved in 95% EtOH and stored at -80 °C until required. Genomic DNA was extracted from thoracic muscle of the specimen using MasterPure™ Complete DNA and RNA Purification Kit (Epicentre Biotechnologies, www.epibio.com). Genomic DNAs with concentrations higher than 200 ng/µL were diluted two—fold with ddH_2_O and used as templates for PCR amplification. Approximately 500 bp fragment of the mitochondrial cytochrome oxidase subunit II gene (*cox2*) was amplified using C2-J-3102 (Jordan et al. 2003) and an *Euphaea*—specific primer, *Euphaea*-C2-N-3740 (5′-TCA TCT AGT GAG GCT TCA-3′) designed by comparing *cox2* sequence variation among *Euphaea* species (Lin et al. 2010; Huang and Lin 2011). Each PCR reaction contained 1 µL of genomic DNA (100 to 300 ng/µL), 1 µL of ProTaq polymerase (2u/µL, Protech Technology, www.protech-bio.com), 2 µL of forward and reverse primer (10 mM), 4 µL of dNTPs (1 mM), 5 µL, of ProTaq buffer, and 35 µL of ddH_2_O. The PCR procedure was as follows: one minute of denaturation at 94 °C, one minute of denaturation at 94 °C followed by 45 seconds of annealing at 53 °C, one minute of extension at 72 °C (repeated 35 cycles), and 10 minutes of final extension at 72 °C. The target PCR products were gel— purified and extracted using a Gel/PCR DNA Fragments Extraction Kit (Geneaid, www.geneaid.com), and then sequenced from both directions using an ABI PRISM™ 377 automatic sequencer (PerkinElmer, www.perkinelmer.com) at Mission Biotech in Taipei, Taiwan. The chromatographs of *cox2* sequences were manually examined for ambiguous base calling. DNA sequences used in this study were deposited in GenBank (Table 1). The sequence alignment and associated phylogenetic trees were submitted to the TreeBASE (ID: 11499).

**Table 1.**
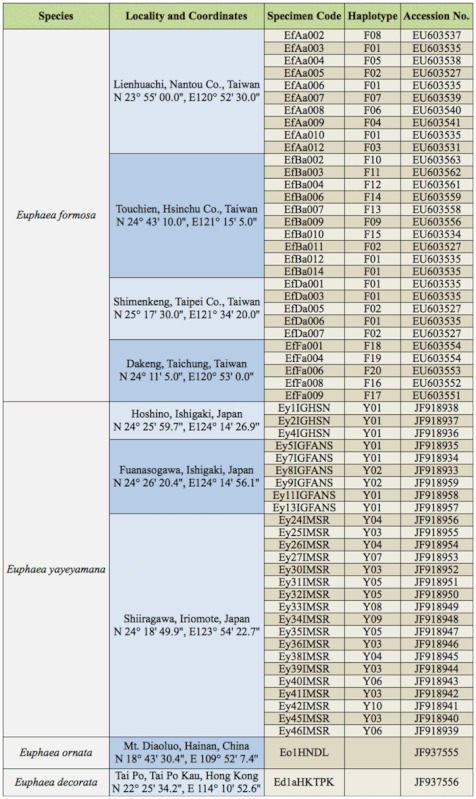
List of analyzed *Euphaea* specimens and their localities and GenBank accession numbers.

### Neutrality test and phylogenetic analyses

DNA sequences were aligned using the Clustal W method in MegAlign (DNASTAR, www.dnastar.com). The McDonald Kreitman Test (MKT) implemented in DnaSP v. 4.0 (Rozas et al. 2003) was used to detect the signature of natural selection in *cox2* by comparing proportions of synonymous and nonsynonymous substitution within vs. between populations. The aligned *cox2* sequences were translated into amino acid sequences in DnaSP using a genetic code of the *Drosophila*. The significance of deviations on the ratio of replacement to synonymous substitutions was determined using two—tailed Fisher's exact tests. For maximum parsimony (MP) analyses, the most parsimonious trees were searched using parsimony ratchet procedure (Nixon 1999) implemented in Pauprat (Sikes and Lewis 2001) and PAUP* v. 4.0b10 (Swofford 2002). The ratchet procedure was run 20 times using 200 replicates in each run and repeated with 15% of weighted characters using batch files implemented in Pauprat. Branch supports were calculated using non—parametric parsimony bootstrapping with 1000 iterations, each with 100 stepwise random sequence additions and tree—bisection and reconnection (TBR) branch swapping. For maximum likelihood (ML) and Bayesian inference (BI) analyses, the best—fitted nucleotide substitution model was selected in Modeltest v. 3.7 (Posada and Crandall 1998) using Bayesian Information Criterion (BIC). ML tree searches of 1000 iterations and parameter optimization were performed using a rapid approximation algorithm implemented in RAxML v. 7.03 (Stamatakis 2006) with starting parameter values derived from the best—fitted substitution model. ML bootstrap analyses of 1000 replicates were conducted with a rapid bootstrapping procedure (-f a) and GTRMIXI model in RAxML to accommodate the proportion of invariable sites (I) and rate heterogeneity using a gamma distribution (Γ). MrBayes v. 3.12 (Huelsenbeck and Ronquist 2001) was used to search BI trees and calculated Bayesian posterior probabilities (BPP) of the trees. Prior values of the model parameters in BI analyses were estimated in Modeltest. Two independent Bayesian analyses with random starting trees were run simultaneously with each run containing four Markov Chains. The Markov Chain Monte Carlo (MCMC) processes were run for 1 × 10^7^ generations with a tree sampling frequency of every 1000 generations. MCMC searches were monitored for the convergence of separate runs after the average split frequencies of two runs fell below the value of 0.01, and the convergence diagnostic potential scale reduction factor reached one (Gelman and Rubin 1992). The first 25% of MCMC samples were discarded as burn—in. BPP of the BI trees was calculated using a 50% majority rule tree from the remaining 7500 trees in PAUP^*^. For statistical network analyses, TCS v. 1.21 (Clement et al. 2000) was used to construct a parsimony network with 95% probability of haplotype connection.

### Geometric morphometrics

Recent studies analyzing wing shapes and DNA sequences successfully discriminated morphologically cryptic insect species and populations (Camara et al. 2006; Favret 2009; Marsteller et al. 2009; Valenzuela et al. 2009; Yee et al. 2009). The geometric morphometric method based on landmarks can separate information concerning shape from size and scaling of morphological structures, therefore allowing these structural characters, which are often correlated, to be tested independently (Zelditch et al. 2004; Slice 2007). The right wing of each damselfly was carefully removed from the preserved specimen and mounted on a glass slide with the dorsal side of the wing facing upwards. A ruler with minimum scales of 1 mm was placed on the glass slide to calibrate of the measurement. A Nikon D80 digital camera with 105 mm Micro Nikkor lens f 2.8 (www.nikon.com) mounted on a copy stand was used to photograph the wings at 7–8× magnification, with two white lights projected from 45-degree angles above the slide and one light directly below the slide. Before taking each image, the slide surfaces were manually adjusted with the aid of a gradienter so that they were perpendicular to the camera. Images were saved in JPEG format (300 dpi) and imported into tpsDig (Rohlf 2005) for digitization of landmarks. A series of twelve landmarks for forewings and hind wings were chosen to quantify wing shape variation (Figure 2A). Two additional landmarks 1 mm apart on the reference ruler were digitized to calculate the centroid sizes of wings but not used for shape analyses. Centroid size was used as an estimate of wing size, which represented a surrogate for body size of the damselfly.

The x and y coordinates of the landmarks were digitized on each wing image and converted into TPS format using tpsDig. The TPS files were imported into CoordGen6h of the IMP (Sheets 2004) for subsequent statistical analyses of wing shape. The Procrustes superimposition method was used to remove non—shape variations including scale, position, and orientation differences among specimens, and to extract shape variables among homologous landmarks using a Generalized Least Square (GLS) criterion (Rohlf and Marcus 1993; Zelditch et al. 2004). For morphometric analyses, samples from all populations within each species were pooled because the main purpose of this study was to distinguish between species, and the sample size was insufficient to allow sensitive statistical tests (3–10 samples per site, Table 1). The geometric shape variables obtained from the GLS were used to conduct the Principal Component Analysis (PCA) implemented in PCAGen6p of the IMP for characterizing wing shape differences between species. Anderson's test was used to determine the numbers of statistically significant PCs that discriminate between the two species (Anderson 1984). The consensus wing shape (mean wing shape) of all specimens was compared with a consensus for each of four categories (the forewing and hind wing of two species) to characterize changes of wing shapes. Thin—plate spline deformation grids were generated between each of the four categories and the consensus in PCAGen6p to visualize the level of deformation in wing shapes. Multivariate analyses of covariance (MANCOVA) were performed in SPSS v. 12.0 (Norusis 2005) to statistically evaluate the wing shape differences between species and between forewings and hind wings. The shape variables (uniform components and partial warps) of fore or hind wings were used as dependent fixed variables and the centroid size and population as a covariate. A multivariate regression of wing shape variables against centroid sizes using pooled samples of both species was conducted in TpsRegr v. 1.38 (Rohlf 2011) to test for a linear pattern of wing shape and body size between both species.

### Comparison of morphological and genetic divergence

To assess whether the morphological and molecular variations are associated with geographical distance, the Mantel test implemented in Isolation By Distance, IBD v. 3.15 (Jensen et al. 2005) was utilized. Pairwise geographical distances between specimens were calculated using the GPS coordinates at the sampling localities in the Geographic Distance Matrix Generator v. 1.2.3 (Ersts 2010). Corrected pairwise genetic distances between specimens were calculated using the Tamura-Nei 3 parameter substitution model in MEGA v. 4 (Tamura et al. 2007). The full set of partial warp scores, uniform components, and centroid sizes obtained from PCAGen6p were size—corrected using the regression of each shape component on individual centroid size. The residuals of the regression of each shape component were used to calculate the pairwise Euclidean distance of wing shape in PRIMER v. 5 (Clarke and Warwick 2001). Partial Mantel tests were performed in IBD to investigate if morphological distance (wing shape and size) was a function of genetic distance, while controlling for the effect of geographical distance. Significance levels for the Mantel and partial Mantel tests were assessed against a null distribution generated by 10,000 randomizations of distance matrices. Reduced Major Axis (RMA) regression was used to estimate the slope and intercept of the relationships, with 95% confidence limits being evaluated by 10,000 bootstrapping replicates over independent specimen pairs.

## Results

### Phylogenies and haplotype networks

**Figure 3.**
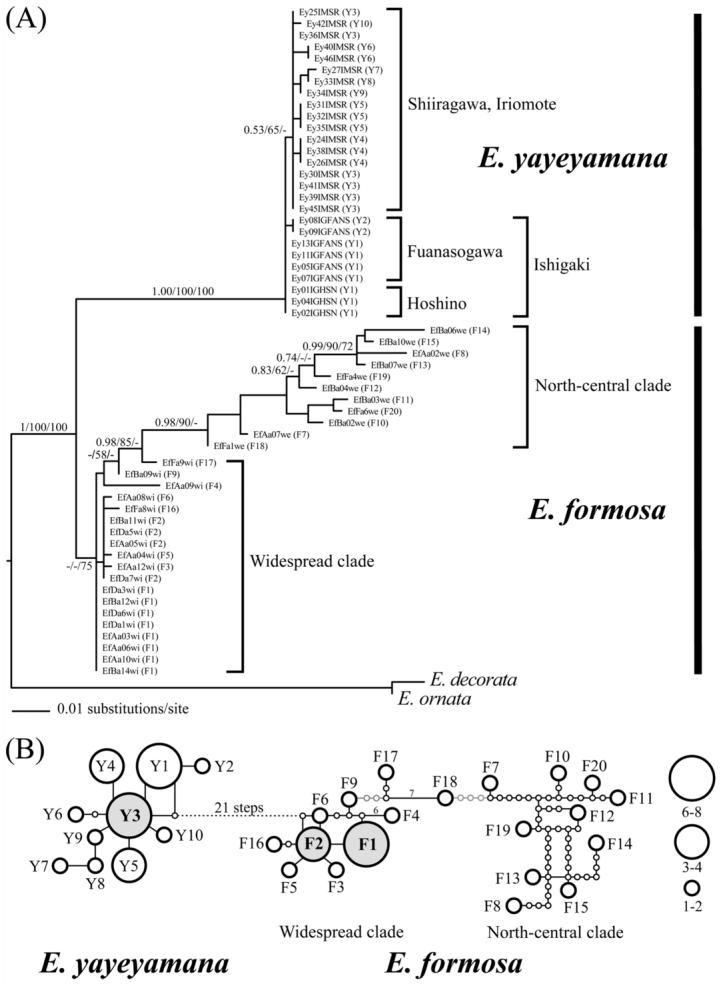
Phylogenetic relationships of *Euphaea formosa* and *Euphaea yayeyamana* based on mitochondrial cox2. (A) 50% majority—rule consensus tree of the Bayesian analyses. Branch support: Bayesian inference/Maximum likelihood/Maximum parsimony. (B) Parsimony haplotype networks with black lines indicating 95% most probable connection and gray lines demonstrating minimum connection steps required to connect all haplotypes of *E. formosa* into a single network. Dashes connect haplotypes that differ by a large number of mutational steps. Gray circles are inferred ancestral haplotypes, and sizes of circles represent the number of individuals carrying particular haplotypes. High quality figures are available online.

Of 57 in—group specimens from seven locations sequenced for *cox2*, 30 haplotypes for *E. formosa* (20, F1–20) and *E. yayeyamana* (10, Y1–10) were identified (Table 1). The sequence alignment of *cox2* was 500 bp and contained 84 variable and 72 parsimoniously informative characters. MKTs had no signs of any selection in *cox2*. One fixed non— synonymous and 17 synonymous substitutions were identified between species, but the ratio of replacement to synonymous substitutions did not significantly deviate from that of neutral expectation (Fisher's exact test, *p* = 0.43). On the basis of corrected genetic distances in the HKY+Γ model, the *cox2* sequences differed at least 5.1±0.1% between the two species. The degree of intraspecific sequence divergences ranged from 0.4±0.1% in *E. yayeyamana* to 3±0.5% in *E. formosa*. However, the range between populations of the two species and their sister taxa, *E. decorata* and *E. ornata*, is from 11±1.7% to 8.3±1.3%. HKY+I+Γ was selected as the best—fit model for ML and BI analyses. The topologies of 4020 equally parsimonious trees (length = 148 steps) obtained from Pauprat analyses and the ML tree (lnL = -1456.046) of RAxML were comparable to the topologies of the BI trees (Figure 3A). The reconstructed Bayesian phylogeny was well resolved to recover the monophyly of *E. formosa* and *E. yayeyamana* with moderate to high branch support. Two distinct *E. formosa* lineages (North—central and widespread clades) were evident on the tree, which is consistent with an earlier genetic study (Huang and Lin 2011). The North—central clade was restricted to northern and central Taiwan, while the widespread clade contained haplotypes that were widely distributed throughout the island. Within *E. yayeyamana*, haplotypes were clustered into Ishigaki and Iriomote clades. The haplotypes from Iriomote assembled into a monophyletic lineage, and that of Ishigaki was paraphyletic with respect to haplotypes of Iriomote. *Cox2* haplotypes separated by up to seven mutational steps were connected into a single network with greater than 95% probability (Figure 3B). Ten mutational steps were required to connect all haplotypes of *E. formosa* into a single network. The TCS analysis placed the haplotype Y3 from Iriomote and the haplotypes F1 and F2 from the widespread clade as ancestral for *E. yayeyamana and E. formosa*, respectively. None of the *cox2* haplotypes was shared between *E. formosa* and *E. yayeyamana*. The haplotypes of *E. yayeyamana* were connected to that of *E. formosa* by 21 mutational steps, indicating that the two species are distantly related genetic lineages.

### Wing shape and body size variation

**Figure 4.**
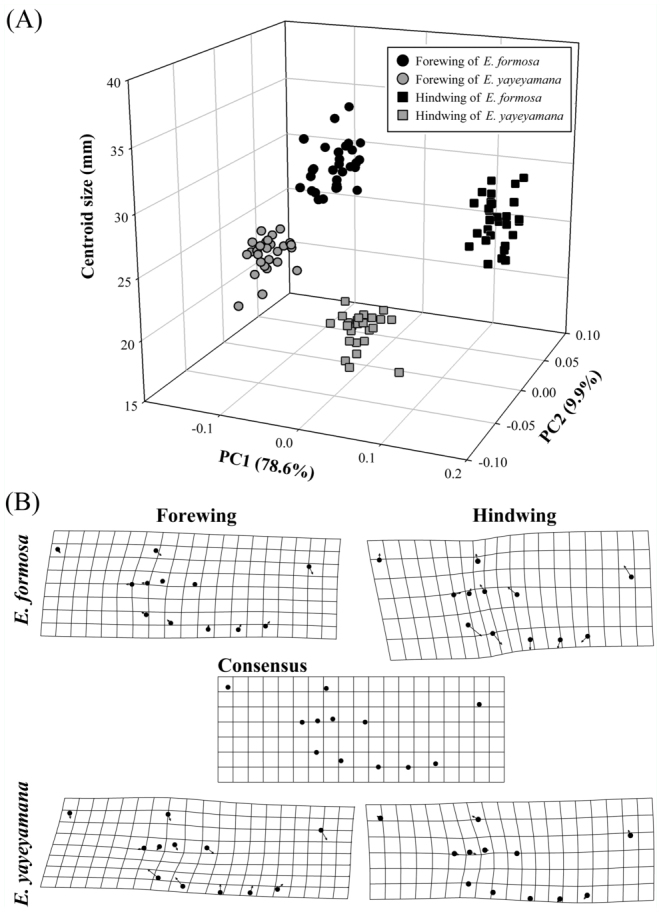
(A) Scatter plot presenting scores on the first two PCs of PCA and centroid sizes for the wings of *Euphaea formosa* and *Euphaea yayeyamana*. (B) Thin—plate spline deformation grids of wing shape variation in *E. formosa* and *E. yayeyamana*, demonstrating the directions (arrows) and amount of deviation from the consensus (mean) wing shape. High quality figures are available online.

The landmark configuration of the Procrustes superimposed coordinates for the wings are presented in Figure 2B. Overall, the landmarks of the hind wing demonstrated more shape variation than that of the forewing, suggested by the areas of scatter of individual landmarks. Landmarks 7, 8, 11, and 12 of the forewing and 1, 3, 8, 9, 10, and 12 of the hind wing are more variable than the other landmarks. Between the two *Euphaea* damselflies, the distribution of all landmark coordinates of the forewing overlapped extensively, whereas the coordinate scatters of the landmarks 6, 7, and 8 of the hind wing had no overlap, suggesting that these positions are more useful for shape discrimination between the two species. Wing shape differed significantly between the two species and between the forewing and hind wing (Table 2). The centroid size also demonstrated a significant effect on wing shape, while the effect of the factor population was negligible (Table 2). The linear correlation between wing shape variables and body was significant (forewing, *Fs* = 17.195, *p* < 0.01; hind wing, *Fs* = 23.617, *p* < 0.01), and the centroid size was responsible for 35.15% (forewing) and 59.69% (hind wing) of wing shape changes in both species. A PCA was performed on all wings to visualize the pattern of shape variation. The first three principal components were found to have distinct eigenvalues (PC1, χ^2^ = 104.35, *p* < 0.01; PC2, χ^2^ = 26.08, *p* < 0.01; PC3, χ^2^ = 12.69, *p* < 0.01; PC4, χ^2^ = 2.03, *p* > 0.25), and each comprised 78.6 (PC1), 9.9 (PC2), and 3.7% (PC3) of total shape variation. A plot of PC1 and PC2 demonstrated no overlapping of wing shapes between the two species or between the forewing and hind wing within species (Figure 4A). Species—specific differentiation was evident in both forewing and hind wing shapes (Figure 4B). *Euphaea formosa* presented broader hind wings, whereas *E. yayeyamana* had narrower forewings. In both species, the hind wing had a wider posterior margin and the forewing a narrower anal area and elongated apex (Figure 4B).

**Table 2.**
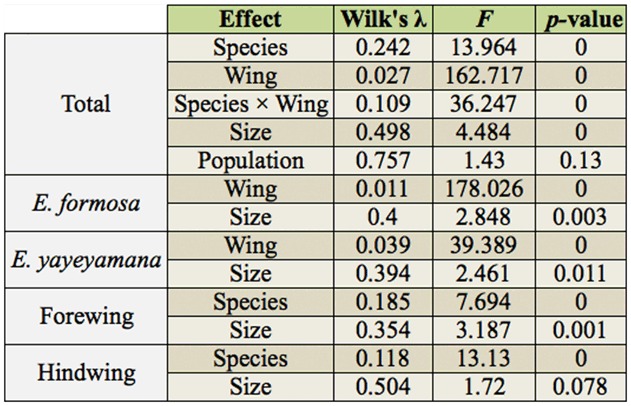
MANCOVA on wing shapes for species (*Euphaea formosa* vs. *Euphaea yayeyamana*) and wing (forewing vs. hind wing).

### Relationships among morphology, genetic divergence, and geographic distance

**Figure 5.**
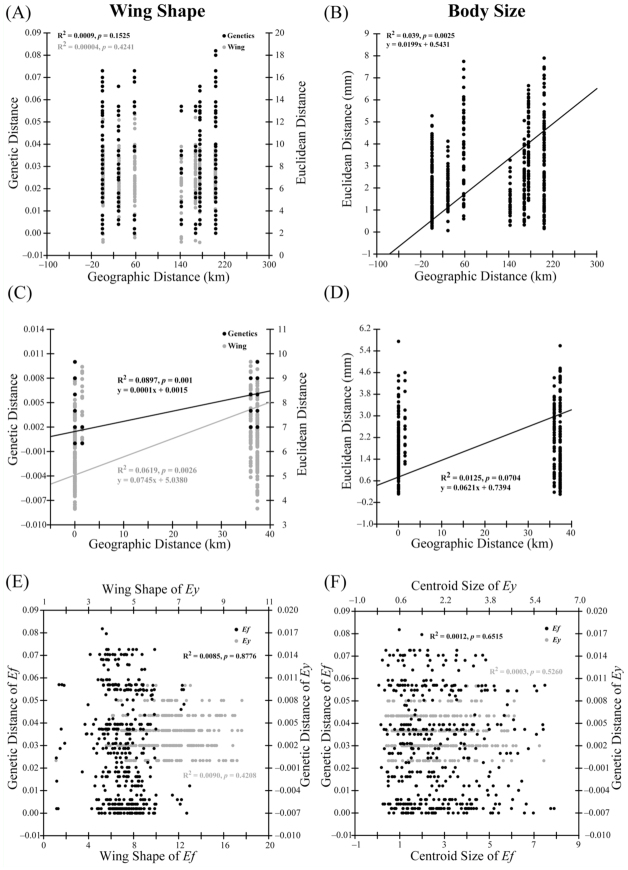
Pairwise plots of morphological vs. geographic distances and genetic vs. geographic distances for *Euphaea formosa* (A and B) and *Euphaea yayeyamana* (C and D), demonstrating the slopes of the RMA regression including their equations and correlation coefficients (R^2^) for each comparison. Pairwise plots of partial correlation between wing shape vs. genetic distances (E) and between body sizes vs. genetic distances (F) after controlling for the effect of geographical distances. *Ef* = *E. formosa, Ey* = *E. yayeyamana*. High quality figures are available online.

Mantel tests measuring the level of correlation between size—corrected morphological (Euclidean distances) and geographical distances (km) were significant in both species (Figures 5A–D), except that the wing shape and geographic distance were not correlated in *E. formosa* (Figure 5A). These correlations indicated a morphological pattern of isolation by distance. Genetic distances between populations were significantly correlated with the geographical distances in *E. yayeyamana* (Figure 5C), but not in *E. formosa* (Figure 5A). After controlling the geographical distance as an indicator variable in partial Mantel tests, the pairwise plots of morphological differentiations and genetic distances demonstrated no significant relationship for either species (Figures 5E and 5F).

### Discussion

This study provides comparative evidence to suggest that the within— and between—species variation in body size and wing shape of these two *Euphaea* damselflies is likely to have an adaptive origin. The divergent body size and wing shape in natural populations may represent adaptation to island environments or habitat heterogeneity. After the geographical effect is taken into account, the evolutionary divergence of size and wing morphology among *E. formosa* and *E. yayeyamana* populations does not follow the expected pattern of neutral evolution indicated by the genetic variation of the *cox2* gene. This indicates that body size and wing shape variations in these two *Euphaea* are most likely the subject of natural or sexual selection for fitness optimization. Stochastic fluctuations in phenotypic traits generated purely by mutation and genetic drift are unlikely to produce a significant correlation between trait values and geography (i.e., environment), as revealed in this study. However, the genetic analyses suggested a past genetic bottleneck in *E. yayeyamana*, with effect of genetic drift in generating trait divergence in these two *Euphaea* species being a possibility. The results of geometric morphometric analyses indicated that there was a linear correlation of wing shape and body size between both species. Since the initial divergence of the two insular species, *E. formosa* has kept a larger body size and broader hind wings, while *E. yayeyamana* has become a much smaller damselfly with elongated forewings and narrower hind wings. These phenotypic adaptations are most likely to have been shaped and maintained by ecological factors associated with habitat differences between the islands. The smaller body size of *E. yayeyamana* represents an insular adaptation to the lower availability of larval prey on Iriomote and Ishigaki Islands than in Taiwan (Hayashi 1990). The reduced body size in *E. yayeyamana* was achieved by decreasing the size of the early instar larvae without changing the number of molts (Hayashi 1990). However, the ecological and evolutionary factors contributing to elongated forewings and narrower hind wings of *E. yayeyamana* inhabiting smaller islands are less well understood. Studies have suggested that various selective pressures including landscape structure (Taylor and Merriam 1995), food and predation stress (Stoks 2001; Svensson and Friberg 2007), and latitude and sexual selection (Outomuro and Johansson 2011) can affect the evolution of wing shapes in damselflies. For *E. yayeyamana*, one possible ecological driver for wing shape evolution is the advantage of resource allocation in the limited available habitats (smaller and shorter forest streams) of smaller Iriomote and Ishigaki Islands (Hayashi 1990), where elongated forewings and narrower hind wings were selected indirectly for covarying smaller body size to optimize resource allocation for lower prey abundance. Another possible source of selection for *E. yayeyamana* wing shape may result from a reduced level of intraspecific sexual selection among males in smaller islands, resulting in wings with lower energy consumption and flight maneuverability. Observations suggested that the abundance and population density of territorial males in *E. yayeyamana* are lower than *E. formosa* in Taiwan (Hayashi 1990; Huang and Lin 2011), suggesting a decreased level of territorial competition. Further studies concerning the ecological and social environments of these two species are necessary to draw conclusions regarding the relative importance of natural versus sexual selection in the evolutionary divergence of wing shapes.

The finding that wing shape differs between the two species supports the hypothesis that the two *Euphaea* damselflies are morphologically distinct. The majority of the wing shape variation between the two species was explained by between—species differences, but the differences between the forewing and hind wing accounted for an important percentage of overall shape variation. The landmark—based wing shape analysis may be useful for discriminating other sibling gossamer—wing damselflies in the *Euphaea* species group of uncertain or puzzling status, such as among the *E. guerini* species complex and geographical populations of *E. masoni* on the mainland of Southeast Asia (van Tol and Rozendaal 1995; Hämäläinen and Karube 2001; Toan et al. 2011), or between *E. subcostalis* and *E. subnodalis* in Borneo (Orr and Hämäläinen 2003). Apparent wing deformation due to damage from emergence or flying activities was observed in some specimens in this study, indicating a need for caution when examining suitable individuals and applying geometric morphometrics of wing shape for species diagnosis. Using wing shape as a discriminating character has an advantage, in that wings are practically two—dimensional structures, making alignment of specimens for digitizing landmarks easier and more accurate than other three—dimensional structural characters, where measuring errors caused by different alignments of individual specimens may constitute a substantial proportion of shape variation (Zelditch et al. 2004). In addition to species discrimination, wing shape analysis was successfully used in recent damselfly studies to investigate population differentiation (European *Calopteryx splendens*, Sadeghi et al. 2009), variation in flight morphology (*Enallagma cyathigerum*, Bots et al. 2009), wing shape evolution (Johansson et al. 2009), and the effects of latitude and selection on wing shape (*Calopteryx virgo meridionalis*, Outomuro and Johansson 2011). Geometric morphometric analysis of wing shape is a useful tool and can be applied to ecological and evolutionary research in odonates (Córdoba-Aguilar 2008).

The mitochondrial *cox2* region indicated that *E. formosa* and *E. yayeyamana* have significant genetic differences, although the two species are morphologically very similar. The sequence divergences within and between *E. formosa* and *E. yayeyamana* are much less than those between their sister taxa, suggesting that they constitute distinct “genetic species” (Mallet 1995). The results of phylogenetic analyses demonstrated that *E. formosa* and *E. yayeyamana* are monophyletic lineages and therefore form true “phylogenetic species” (de Queiroz and Donoghue 1988). In addition to the recognition of distinct species, the phylogenetic analyses revealed the presence of substantial genetic structure within *E. formosa*, where a North—central clade with a balanced tree topology was restricted to northern and central Taiwan, and a star—like widespread clade was widely distributed throughout the island. An earlier study concerning extensive genetic sampling of *E. formosa* indicated that the North—central clade maintained a slowly growing population, whereas the widespread clade experienced a spatial and demographic expansion into eastern Taiwan (Huang and Lin 2011). In this study, the phylogenetic results indicated that the present *E. yayeyamana* demonstrates little genetic differentiation and no phylogeographical substructure, with the exception of the separation of haplotype clusters between Iriomote and Ishigaki Islands. The shallow tree topology and low genetic differentiation indicated that the island populations of *E. yayeyamana* are descendants of a few founders from Taiwan and may have experienced a severe genetic bottleneck or population expansion in recent history. The lower haplotype diversity of *E. yayeyamana* on Iriomote and Ishigaki Islands may be due to a smaller effective population size, resulting in a greater effect of genetic drift than in the larger *E. formosa* populations in Taiwan.
